# GWAS combined with linkage analysis reveals major QTLs and candidate genes of salt tolerance in *Japonica* rice seedlings

**DOI:** 10.3389/fpls.2024.1462856

**Published:** 2024-11-01

**Authors:** Shanbin Xu, Jie Zheng, Haoqiang Du, Xiaodong Du, Chong Li, Yuxuan Duan, Yanan Cai, Jingguo Wang, Hualong Liu, Luomiao Yang, Wei Xin, Yan Jia, Detang Zou, Hongliang Zheng

**Affiliations:** ^1^ Key Laboratory of Germplasm Enhancement and Physiology & Ecology of Food Crop in Cold Region, Ministry of Education/College of Agriculture, Northeast Agricultural University, Harbin, China; ^2^ Rice Research Institute, Heilongjiang Academy of Agricultural Sciences, Jiamusi, China

**Keywords:** *Japonica* rice seedling, salt tolerance, QTL, candidate gene, GWAS, linkage analysis

## Abstract

**Background:**

Soil salinization is one of the significant factors limiting high crop yields and expansion of arable land, seriously affecting global agricultural production. Rice is an essential food crop throughout the world, and its seedlings are particularly susceptible to salt stress, which can directly affect the growth and development of rice and its final yield. We used the natural population as the material for genome-wide association study (GWAS) and the recombinant inbred line (RIL) population from CD (salt sensitive)/WD20342 (salt tolerant) hybridization as the material for linkage analysis.

**Results:**

The degree of salt tolerance was evaluated by using the relative root length (RRL), relative root number (RRN), relative root fresh weight (RRFW), and relative root dry weight (RRDW) as indicators. Fifteen and six major quantitative trait loci (QTLs) were identified by GWAS and linkage analysis, respectively. Meanwhile, the GWAS identified the lead SNP (Chr2_22340368), which was located within qRRL2 and qRRDW2 identified by linkage analysis. GWAS, combined with linkage analysis, selected a 196-kb overlapping region on chromosome 2, including 22 candidate genes. *LOC_Os02g36880* was discovered as the candidate gene involved in salt tolerance by haplotype analysis, qRT-PCR, and sequence analysis. The score of salinity toxicity (SST) and seedling survival rate (SSR) were determined for CRISPR/Cas9 mutants (CR-1 and CR-15) and wild-type (ZH11), respectively.

**Conclusion:**

The phenotypic validation indicated that *LOC_Os02g36880* negatively regulated the salt tolerance at the seedling stage. This study provides resources for breeding Japonica rice to improve its response to salt stress.

## 1 Introduction

Rice is an essential crop globally, and with the growing global population, the demand for higher rice yields is increasingly urgent. However, various abiotic factors including drought, salinity, alkalinity, and cold, significant impact crop yields (Radha et al., 2023; Raj and Nadarajah, 2023; Sarma et al., 2023). Among these, salt damage is one of the most critical factors reducing rice productivity, as rice is highly sensitive to saline conditions (Ruan et al., 2011; Chen et al., 2022). Soil salinity affects a substantial portion of arable land, and the shrinking availability of agricultural land further constrains global agricultural productivity. High salt concentrations lead to ionic toxicity, hyperosmotic stress, and secondary stresses, which together disrupt essential rice processes throughout its life cycle, from germination to senescence (Hanin et al., 2016; Zhu, 2016). Rice is particularly vulnerable to the adverse effects of salt stress during the seedling stages. Thus, understanding the genetic mechanisms and identifying relevant QTLs or genes is crucial for developing salt-tolerant rice cultivars, which forms the theoretical and practical foundation for breeding varieties.

Salt tolerance in rice is a typical quantitative trait jointly controlled by multiple genes and the integrated expression of various mechanisms (Liu et al., 2022; Phosuwan et al., 2024). Significant progress has been made by employing forward genetics strategies, such as GWAS and linkage analysis, to identify numerous QTLs and genes associated with salt tolerance. For instance, Kumar et al. (2015) evaluated twelve salt-related agronomic traits using a natural population of 220 rice accessions. By combining these traits with a gene microarray containing 6,000 single nucleotide polymorphisms (SNPs), they identified sixty-four loci, which explained 5% to 18% of the phenotypic variance. Similarly, Al-Tamimi et al. (2016) conducted a GWAS on 553 rice varieties identified 12 efficient QTLs associated with salt tolerance based on indicators such as relative growth rate, transpiration rate, and transpiration efficiency. Batayeva et al. (2018) identified 26 QTLs linked to salt tolerance in 203 rice accessions, with eleven of these QTLs overlapping with previously known loci. Rohila et al. (2019) used a hydroponic system to subject 162 rice accessions to salt stress during the seedling stage, leading to the identification of nine genomic regions associated with salt tolerance by analyzing over three million SNPs. Additionally, they discovered six new loci and identified sixteen potential genes. Yuan et al. (2020) utilized 664 cultivated rice materials for GWAS, identifying 21 QTLs associated with salt tolerance.

Meanwhile, researchers have also found several QTLs using linkage analysis. De Leon et al. (2016) utilized a RIL population derived from a cross between the Bengal (salt-sensitive) and the Pokkali (salt-tolerant) to identify 85 additive QTLs for nine salt-related traits, including salt injury score, shoot length, root length. Nakhla et al. (2021) identified 23 and 46 QTLs controlling salt tolerance at the germination and seedling stages, respectively, through linkage mapping. Zeng et al. (2021) used the a BC_1_F_2_ population derived from a cross between WJZ and Nipponbare to identify the major salt tolerance QTL *qGR6.2*, during the germination stage and predicted 11 candidate genes. Geng et al. (2023) employed a RIL population derived from a cross between Milyang 23 (salt-tolerant) and Jileng 1 (salt-sensitive) to identify 12 QTLs through linkage mapping.

In this study, we explored the genetic foundation of salt tolerance in rice using GWAS and linkage analysis with RRL, RRN, RRFW, and RRDW during the rice seedling salt treatment. Both GWAS and linkage analysis are powerful tools for detecting QTLs associated with complex traits, and their combination can significantly enhance the accuracy of QTL detection. Our GWAS identified a lead SNP (Chr2_22340368), within the QTLs *qRRL2* and *qRRDW2*, which were also identified through linkage analysis. A 196-kb region on chromosome 2 was selected for further examination based on the overlapping regions identified through both GWAS and linkage mapping. Knockout mutants were subsequently used for the functional verification, leading to the identification of *LOC_Os02g36880* as a gene that contributes to salinity resistance.

## 2 Materials and methods

### 2.1 Plant materials

The natural population was conducted using the 295 *Japonica* rice materials gathered from Chinese provinces such as Heilongjiang, Ningxia, and Jilin, along with foreign varieties mainly from Japan, Russia, and Korea (Li et al., 2019). The linkage mapping population consisted of 189 RILs, a cross between the CD (salt-sensitive) and WD20342 (salt-tolerant) (Li et al., 2023). **Supplementary Figure S1** shows CD and WD20342 salt tolerance phenotypes. In Heilongjiang Province, China, at the Northeast Agricultural University Experimental Station (45°52′N, 127°04′E), all materials were planted.

### 2.2 Phenotype data

All materials were baked for 48 hours at 55°C, after choosing and disinfecting the seeds for 20 minutes with a 3% NaClO solution. The seeds were cultured hydroponically at 30°C for 4 days. Using the Yoshida nutrient solution, sixty-four evenly germinated seedlings of each type were divided into salt-treated and control groups for hydroponic cultivation. The germinated seeds were incubated at 26 and 24°C, with a light and dark cycle of 14 and 10 hours, respectively. Once the seedlings reached two leaves and one heart, the control group received standard nutrient solution application; the treatment group received salt stress treatment. Pretreatment of the seedlings was 50 mM NaCl, which was applied for 3 days, and formally treated with 120 mM NaCl for 7 days. Five plants were randomly chosen for the measurement in each material, including the root length, the root number, the root fresh weight, and the root dry weight under control and salt treatment. Further calculations were performed to determine the RRL, RRN, RRFW, and RRDW: the relative values represent the ratio of phenotypic values during salt treatments to phenotypic values under control conditions. Three replications of the experiment were set up.

### 2.3 GWAS

Using Plink 2.0 software (Chang et al., 2015), 788,396 SNPs were identified with a minor allele frequency (MAF) of ≥5% and a missing rate of ≤20%. The 295 materials were analysed by GWAS using 788,396 SNPs. The calculated population structure (Q) and kinship between individuals were used to adjust the population structure of GWAS (Li et al., 2019). The mixed linear model (MLM) included in TASSEL 5.0 (Bradbury et al., 2007) was used for GWAS. The threshold for the identification of SNPs significantly associated with traits was set to –log_10_(*P*) > 5.26, determined by the genetic type 1 error calculator (GEC; http://statgenpro.psychiatry.hku.hk/gec/), which calculates the effective number of independent markers. Generate the Manhattan map using the R program ‘qqman’. To determine the leading SNPs with the lowest P-value, redundant SNPs were filtered using a least-distance interval. Utilizing the LDBlockShow software, the pairwise *R^2^* value between any two SNPs within the interval of leading SNPs ± 2 Mb was determined. The LD attenuation interval of leading SNPs between 1.5 and 2.0 Mb was the average of the top 10% *R^2^* values plus 0.2 (Dong et al., 2020).

### 2.4 Linkage analysis

Using 10 K Array targeted sequencing technology from MOLBREEDING Biotechnology Company, the genotypes of the RIL lines were determined. Following biparental polymorphism analysis and de-redundant analysis and by the IciMapping-Bin project, there was a grouping of specific SNP markers with the same genotype into one “BIN”. Using 189 RILs from CD and WD20342 and 978 bin markers, a genetic linkage mapping was produced. With an average distance of 2.52 cM between markers, the map contains 2465.33 cm of the genome (**Supplementary Figure S2**). The inclusive composite interval mapping method used in ICIMapping Ver.4.2 was utilized for linkage analysis, with LOD > 2.5 as the threshold value (Lei et al., 2021).

### 2.5 Haplotype analysis

Non-synonymous mutant SNPs in the promoter (1.5 kb before ATG) and exonic regions of genes were retrieved using the RiceSNPSeekDatabase website (https://snp-seek.irri.org/_snp.zul). Haplotype analysis was done by the DnaSP software application. The number of materials needs to be ten or more to be classified as different haplotypes.

### 2.6 qRT-PCR and sequence analysis

Using qRT-PCR analysis in both standard and salinized conditions, the expression levels of the candidate genes associated with both CD and WD20342 were confirmed. Roche LightCycler96 was used to carry out the qRT-PCR analysis. **Supplementary Table S3** contains all of the primer sequences. The promoter and exonic regions of genes were amplified using PCR.

### 2.7 *LOC_Os02g36880* mutant plants

By utilizing CRISPR/Cas9 technology, the mutants were created, and the T1 generation *LOC_Os02g36880* knockout mutant seeds in the Zhonghua11 (ZH11) background were made by BIOGLE GENETECH company (http://www.biogle.cn/). PCR amplification was used to identify two homozygous mutants (CR-1, CR-15) in the T1 generation. These plants were then selfed to produce the T3 generation. The experiment was conducted with three replications using the wild-type (ZH11) and T3 generation homozygous mutants as the experimental materials. The experimental materials were separated into two groups. One group got a regular nutrient solution when they reached two leaves and one heart, while the other group received a nutrient solution with 120 mM NaCl. The therapy lasted 7 days, followed by a recovery period of 10 days. The treatment lasted for 7 days, and the recovery period lasted for 10 days. Salt tolerance was determined by measuring the SST and SSR.

## 3 Results

### 3.1 Phenotypes of GWAS and RIL population

**Figure 1:**
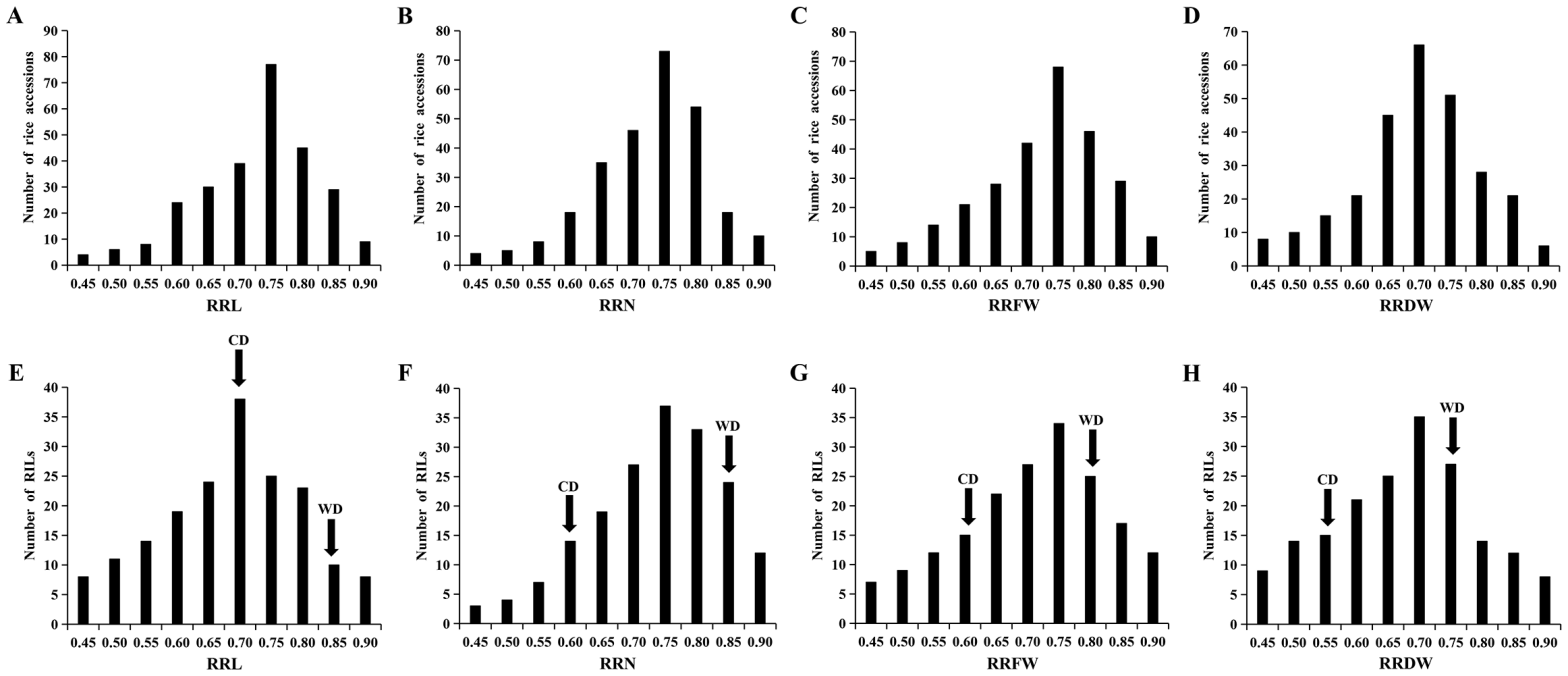
Phenotypic variation in the RRL, RRN, RRFW, and RRDW. **(A-D)** The RRL, RRN, RRFW, and RRDW of 295 materials. **(E-H)** The RRL, RRN, RRFW, and RRDW of RIL lines.

Data from 295 *Japonica* rice materials and RIL lines were analyzed statistically to determine four salt tolerance phenotypes: RRL, RRN, RRFW, and RRDW. Under the salt stress during the seedling stage, the RRL, RRN, RRFW, and RRDW among the 295 materials varied in range from 0.39-0.88, 0.41-0.88, 0.38-0.87, and 0.39-0.87, respectively, with corresponding coefficients of variation of 13.65%, 12.79%, 14.74%, and 14.71% (**Supplementary Table S1**). The ranges of variation for RRL, RRN, RRFW, and RRDW in the RIL lines were 0.37-0.87, 0.44-0.88, 0.40-0.89, and 0.39-0.89, with the coefficients of variation of 17.05%, 13.89%, 17.22%, and 17.78%, respectively (**Supplementary Table S1**). The phenotypic data demonstrate a wide range of phenotypic diversity to *Japonica* rice seedling salt tolerance among the 295 materials and RIL lines. The phenotypic values of four traits significantly differed between WD20342 and CD, revealing that WD20342 was more salinity-tolerant than CD (**Supplementary Table S1**). The RRL, RRN, RRFW, and RRDW phenotypic values of the 295 materials and RIL lines were typically distributed and showed continuous phenotypic variation for each trait, suggesting that these traits are quantitative and affected by multiple factors (**Figure 1**).

### 3.2 GWAS of salt-related traits in the seedling stage

**Figure 2:**
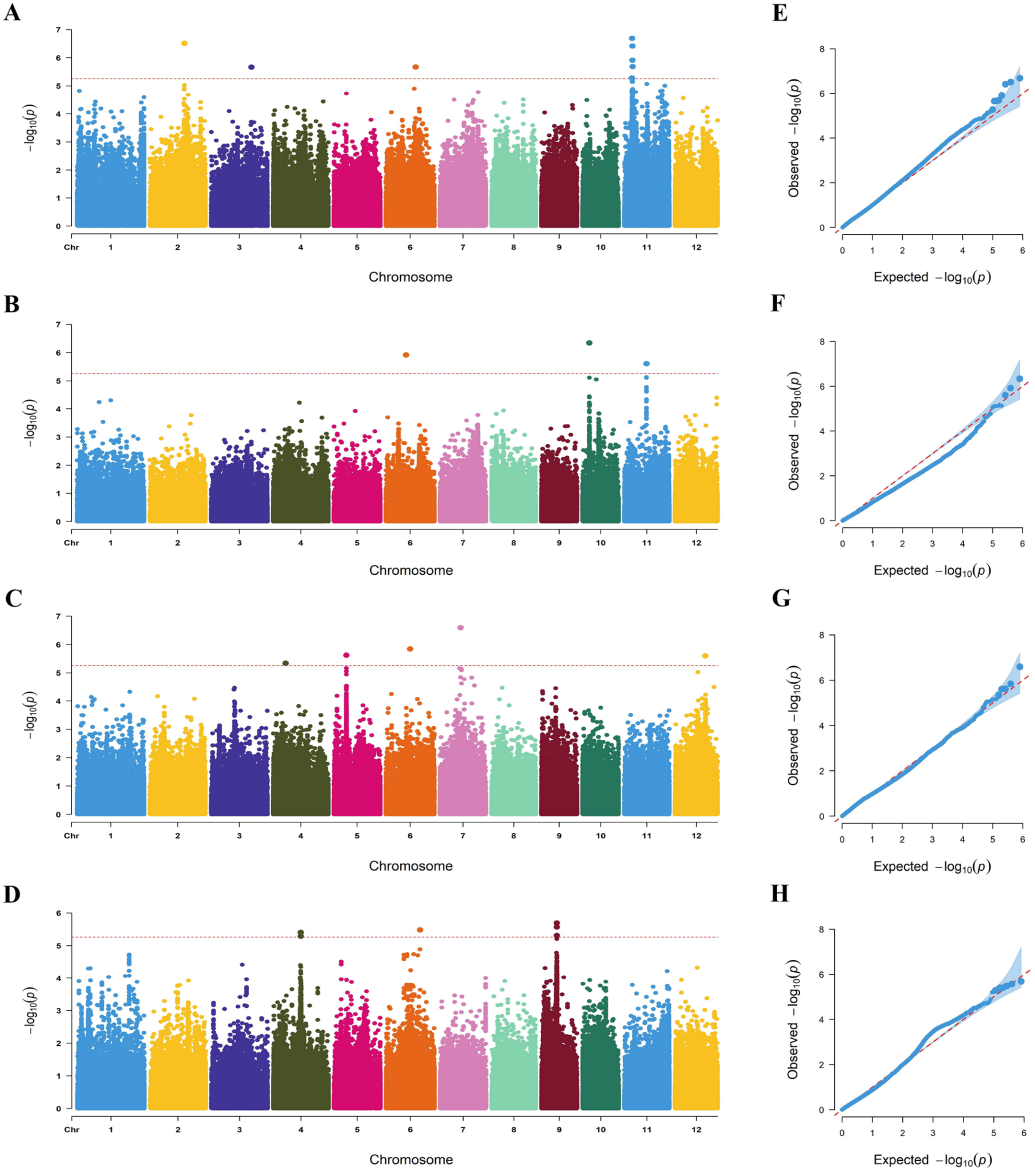
Manhattan plots and Q-Q plots. **(A-D)** Manhattan plot for the RRL, RRN, RRFW, and RRDW. **(E-H)** Q-Q plot for the RRL, RRN, RRFW, and RRDW.

GWAS was performed using the 788,396 SNPs acquired from previous research (Duan et al., 2022; Li et al., 2022). Fifteen lead SNPs were significantly related to RRL, RRN, RRFW, and RRDW by GWAS (**Table 1**; **Figure 2**). Four QTLs linked to RRL were identified on chromosomes 2, 3, 6, and 11, with *R^2^* values varying between 9.52% and 11.30%. Three QTLs linked to RRN were identified on chromosomes 6, 10, and 11, with *R^2^* values ranging from 8.35% to 11.12%. Five QTLs linked to RRFW were identified on chromosomes 4, 5, 6, 7, and 12, with *R^2^* values ranging from 10.14% to 12.21%. Meanwhile, three QTLs linked to RRDW were identified on chromosomes 4, 6, and 9, with *R^2^* values ranging from 8.42% to 10.27%.

**Table 1:** Lead SNPs associated with RRL, RRN, RRFW, and RRDW were identified by GWAS.

Traits	Lead SNP	Chromosome	Position	P value	*R^2^* (%)	Known QTLs	Known genes
RRL	Chr2:22340368	2	22340368	3.09E-07	10.98	*qNAK-2* (Yao et al., 2005)	
	Chr3:25974535	3	25974535	2.16E-06	9.52	*qNaLV-3.1* (Ammar et al., 2009)	
	Chr6:19195187	6	19195187	2.13E-06	9.53	*qRFW-6* (Mardani et al., 2014)	
	Chr11:5007312	11	5007312	2.04E-07	11.30	*qDSS11* (Qiu et al., 2015)	
RRN	Chr6:12954487	6	12954487	1.21E-06	8.89	*qRFW-6* (Mardani et al., 2014)	
	Chr10:4241399	10	4241399	4.57E-07	11.12	*qRL-10b* (Atefeh et al., 2013)	
	Chr11:14309848	11	14309848	2.46E-06	8.35	*qDRW11* (Wang et al., 2012)	
RRFW	Chr4:7964143	4	7964143	4.57E-06	11.57		
	Chr5:8072746	5	8072746	2.38E-06	10.51		
	Chr6:15555901	6	15555901	1.44E-06	10.53	*qRFW-6* (Mardani et al., 2014)	
	Chr7:13210353	7	13210353	2.54E-07	12.21		
	Chr12:19562318	12	19562318	2.49E-06	10.14	*qSNC-12* (Zheng et al., 2015)	*OsbHLH133* (Wang et al., 2013)
RRDW	Chr4:17624511	4	17624511	3.92E-06	9.73		
	Chr6:21996964	6	21996964	3.30E-06	8.42	*qDSW6.1* (Wang et al., 2012)	
	Chr9:9824947	9	9824947	2.04E-06	10.27		

*R^2^* (%): Phenotypic variance explained.

### 3.3 QTL mapping of RIL

Six major QTLs were found on chromosomes 1, 2, 6, 10, and 11 in the RIL lines. The explained phenotypic variance varied from 4.82% to 17.26%, while the LOD values ranged from 2.55 to 7.57. There was one QTL associated with RRL (*qRRL2*), one QTL associated with RRN (*qRRN6*), two QTLs associated with RRFW (*qRRFW1* and *qRRFW10*), and two QTLs associated with RRDW (*qRRDW2* and *qRRDW11*) (**Table 2**). Furthermore, *qRRL2* and *qRRDW2* were defined as the same QTL because of the same interval, located in the physical region between markers C2_21864234 and C2_24239570 and explaining 9.38-17.26% of the phenotypic variance.

**Table 2:** QTLs for RRL, RRN, RRFW, and RRDW were identified by linkage analysis.

Traits	QTLs	Left Marker	Right Marker	Chr.	LOD	*R^2^* (%)	Additive Effect	Known QTLs	Known genes
RRL	*qRRL2*	C2_21864234	C2_24239570	2	3.83	9.38	-0.04	*qNAK-2* (Yao et al., 2005)	
RRN	*qRRN6*	C6_29809865	C6_30179619	6	2.78	8.84	-0.03		*OsCYP19-4* (Yoon et al., 2016)
RRFW	*qRRFW1*	C1_42388254	C1_42399585	1	2.55	4.82	-0.03		
	*qRRFW10*	C10_1090799	C10_1185808	10	3.55	7.65	0.04		
RRDW	*qRRDW2*	C2_21864234	C2_24239570	2	7.57	17.26	-0.06	*qNAK-2* (Yao et al., 2005)	
	*qRRDW11*	C11_23355381	C11_23543062	11	4.02	6.33	-0.04		

*R^2^* (%): Phenotypic variance explained.

**Figure 3:**
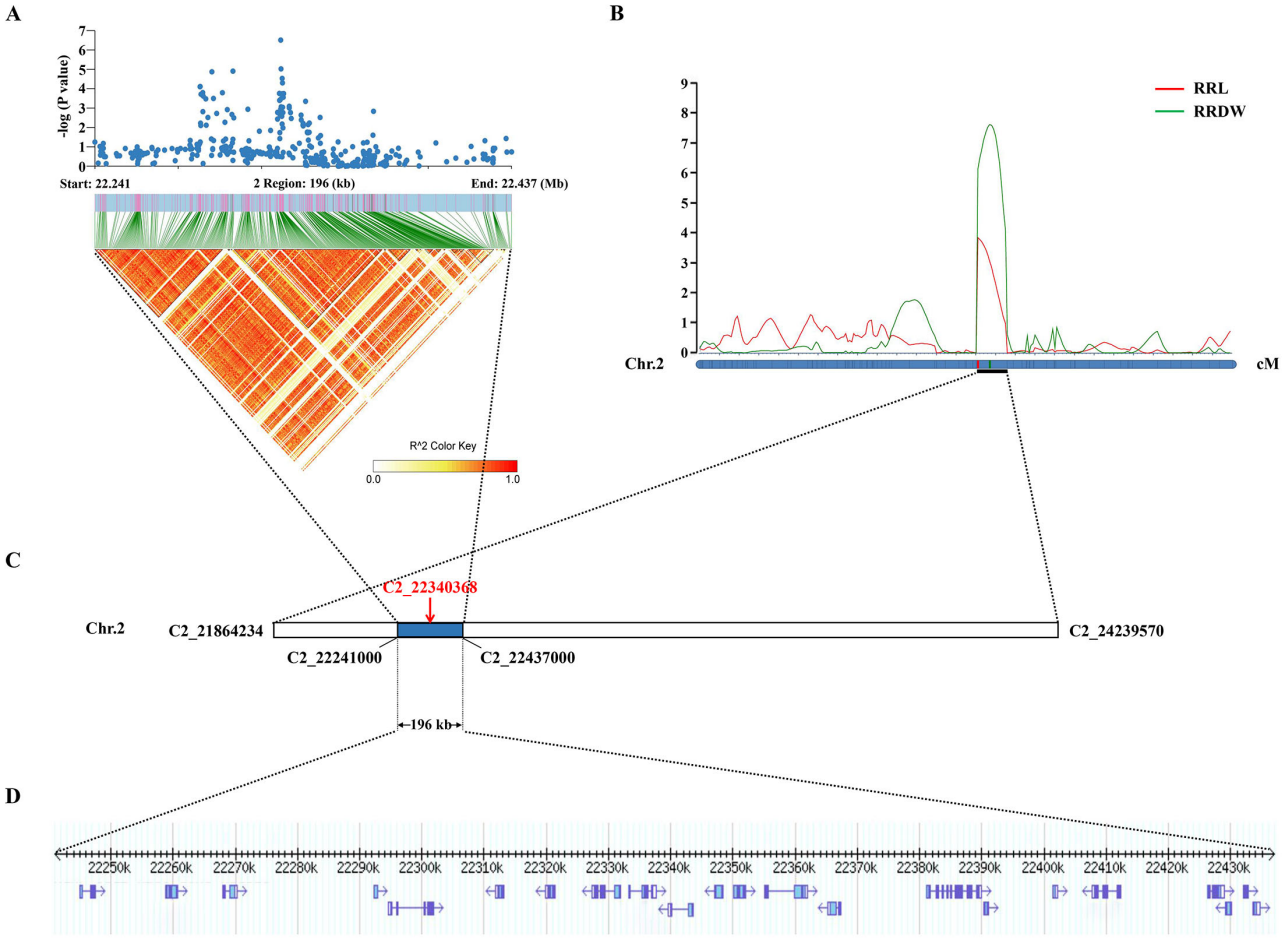
Co-location results of GWAS and linkage analysis. **(A)** The local Manhattan plot and LD heatmap surround the lead SNP. **(B)** Mapped to the interval between markers C2_21864234 and C2_24239570 by linkage analysis. **(C)** The lead SNP (C2_22340368) was identified by the GWAS. **(D)** The 196-kb region.

Significantly, the GWAS discovered the lead SNP related to RRL (Chr2_22340368), which was located within *qRRL2* and *qRRDW2*. On chromosome 2, the LD block region was estimated to be 22.241-22.437 Mb (196 kb). Using GWAS and linkage analysis, a 196-kb overlap region was identified (**Figure 3**). Therefore, we identified the 196-kb overlap region as the significant QTL for candidate gene identification.

### 3.4 Haplotype analysis of candidate genes

**Figure 4:**
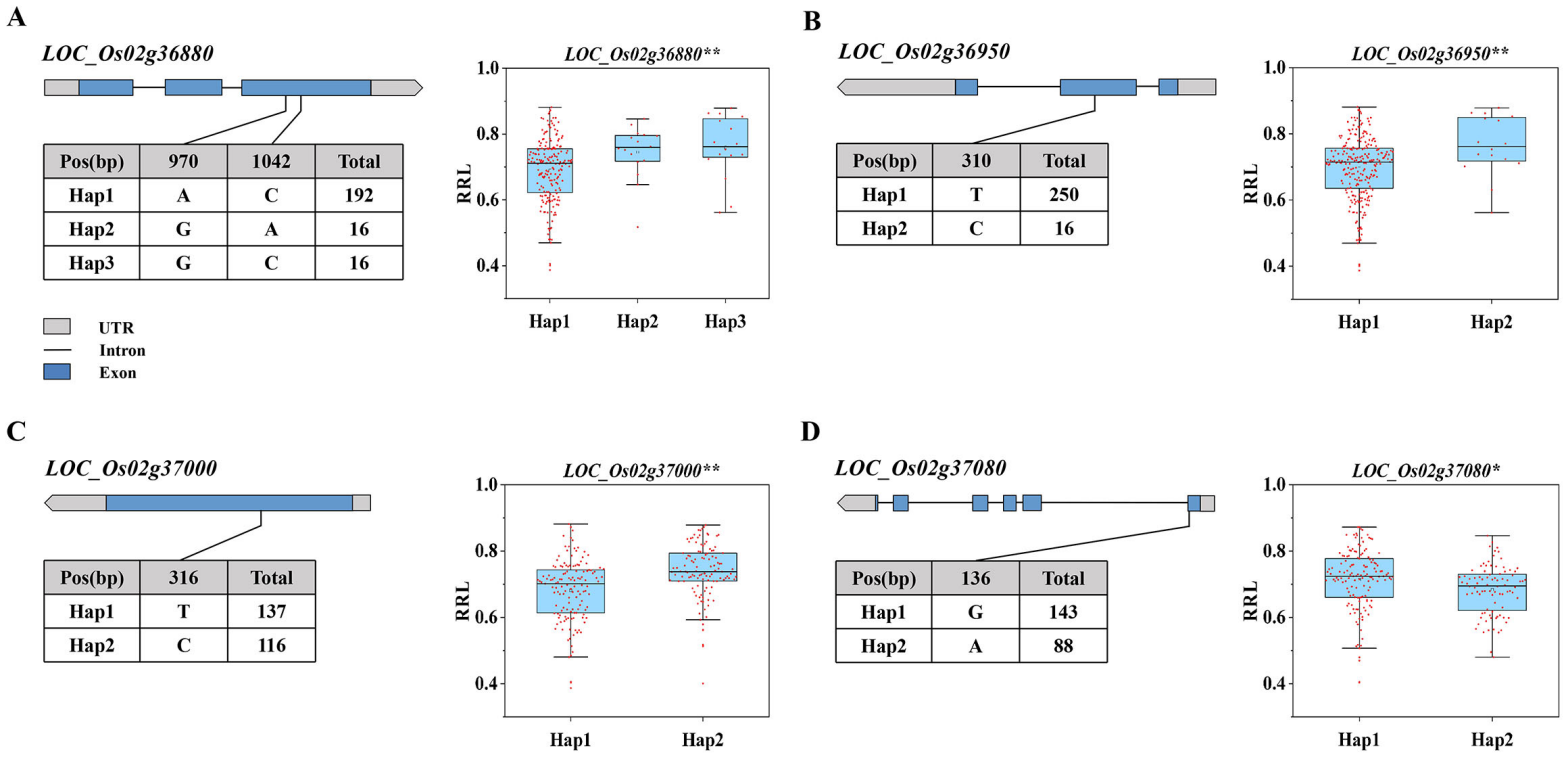
Haplotype analysis of candidate genes. **(A-D)** The haplotype analysis of *LOC_Os02g36880*, *LOC_Os02g36950*, *LOC_Os02g37000*, and *LOC_Os02g37080*. (The * and ** suggest significance of ANOVA at *P* < 0.05 and *P* < 0.01, respectively).

The Rice Genome Annotation Project confirms that the 196-kb region contained 22 genes (**Supplementary Table S2**). We conducted haplotype analysis on 22 genes and identified 4 genes (*LOC_Os02g36880*, *LOC_Os02g36950*, *LOC_Os02g37000*, *LOC_Os02g37080*) in the overlapping region. *LOC_Os02g36880* was classified into three haplotypes based on non-synonymous mutant SNPs in the exon region. *LOC_Os02g36950*, *LOC_Os02g37000*, and *LOC_Os02g37080* were separated into two haplotypes due to non-synonymous mutation SNPs in the exon region (**Figure 4**).

### 3.5 Identification of candidate genes

**Figure 5:**
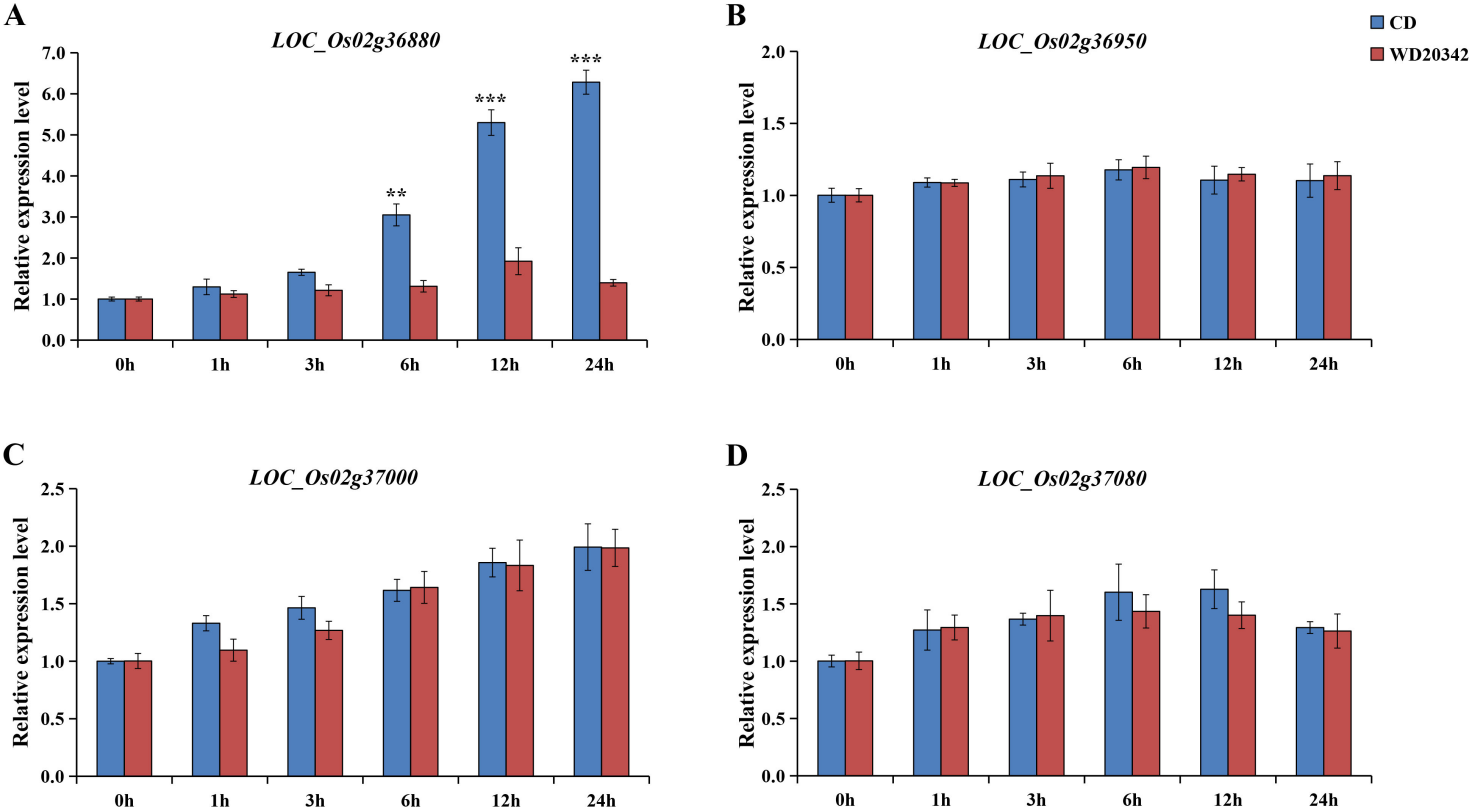
The candidate gene expression patterns were confirmed. **(A-D)** The expression of *LOC_Os02g36880*, *LOC_Os02g36950*, *LOC_Os02g37000*, and *LOC_Os02g37080* genes under control conditions and salt stress was analyzed. (** *P* < 0.01, *** *P* < 0.001, Students’t-test).

Both parents, CD and WD20342, were treated with 120 mM NaCl for 0, 1, 3, 6, 12, and 24 h, respectively. Four genes (*LOC_Os02g36880*, *LOC_Os02g36950*, *LOC_Os02g37000*, *LOC_Os02g37080*) were determined by qRT-PCR analysis. *LOC_Os02g36950* was no induction at all under salt stress, and expression of the other genes (*LOC_Os02g36880*, *LOC_Os02g37000*, and *LOC_Os02g37080*) were up-regulated under stress. Interestingly, only *LOC_Os02g36880* showed significant upregulation of CD and WD20342 expression under salt stress, with significant differences in expression levels. In the 24h salt stress, the expression level of *LOC_Os02g36880* is more than four times higher in CD than WD20342 (**Figure 5**). Meanwhile, qRT-PCR analysis of other functionally annotated genes within the 196-kb region showed that the eight genes expressed between the two parents were not differentially expressed (**Supplementary Figure S3**).

The sequencing of promotor and exonic regions of the genes (*LOC_Os02g36880*, *LOC_Os02g36950*, *LOC_Os02g37000*, and *LOC_Os02g37080*) was conducted for the two parents, CD and WD20342, after analyzing the initial findings. *LOC_Os02g36880* in the CD (salt-sensitive) matches the reference sequence from Nipponbare, as shown by the findings. Contrarily, *LOC_Os02g36880* in WD20342 (salt-tolerant) had one SNP mutation in the promoter region, 6-bp insertion mutations in the first exon region, one SNP mutation in the second exon region, and two SNP mutations in the third exon region (**Supplementary Figure S4**). The three genes (*LOC_Os02g36950*, *LOC_Os02g37000*, and *LOC_Os02g37080*) in CD and WD20342 was not differ regarding mutations. Consequently, *LOC_Os02g36880* was found to be the functional gene in salt tolerance, and its function was further verified.

### 3.6 Validation of the *LOC_Os02g36880* mutant

**Figure 6:**
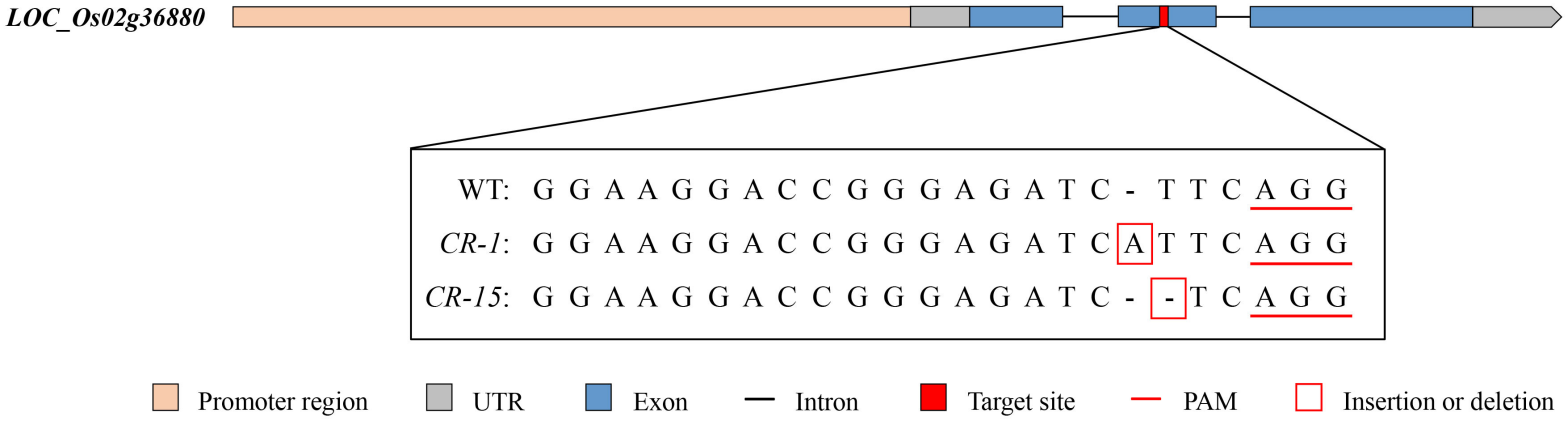
Target sequences of WT (ZH11) and knockout mutants (CR-1, CR-15).

**Figure 7:**
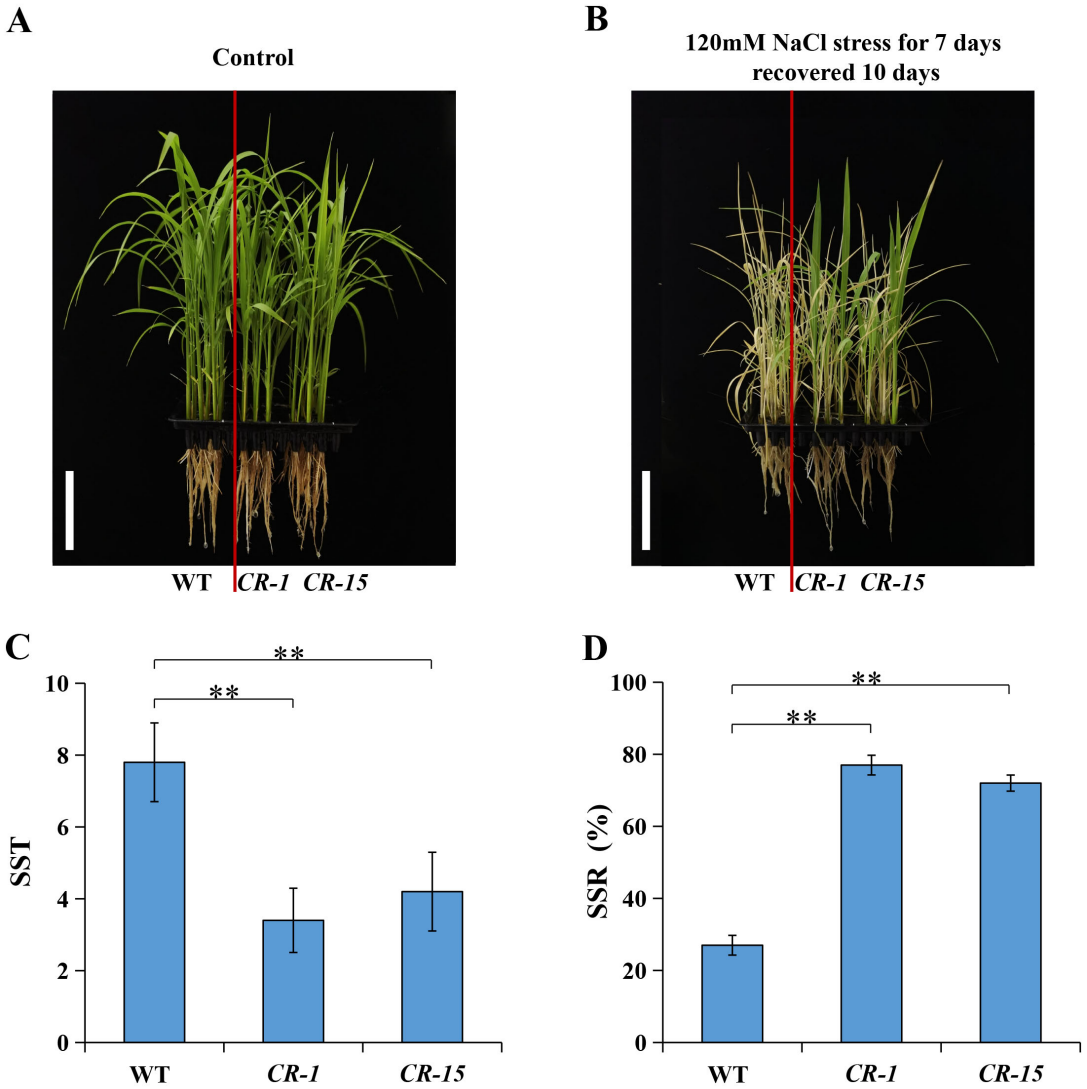
Salt tolerance phenotype of WT and knockout mutants. **(A)** Phenotype of control conditions; **(B)** Phenotype of the salt stress conditions; **(C)** The SST under salt stress; **(D)** The SSR under salt stress. (** *P* < 0.01; Students’ *t*-test).

We selected two homozygous CRISPR/Cas9 mutants, CR-1 with a one-base (A) insertion and CR-15 with a one-base (T) deletion (**Figure 6**) to investigate whether *LOC_Os02g36880* was associated with salt tolerance. These plants were propagated to the T3 generation for further analysis. The analysis found no significant difference in wild-type and CRISPR/Cas9 mutant seedlings under normal conditions. While treated with salt, mutants CR-1 and CR-15 showed higher salt tolerance than wild-type, with average SST values of 3.4 and 4.2, and SSR values of 77.0% and 72.0%, respectively. In contrast, the wild-type was an average SST of 7.8 and an average SSR of 27.0% (**Figure 7**). The findings suggest that the knockout mutant of *LOC_Os02g36880* enhances salt tolerance in seedlings.

## 4 Discussion

The combination of GWAS and linkage analysis to locate gene loci for traits can effectively improve the accuracy and precision of association loci and obtain reliable target intervals. In recent years, this method has been widely applied in genetic studies of rice traits, such as salt tolerance (Kong et al., 2021), alkalinity tolerance (Li et al., 2020), disease resistance (Xiao et al., 2019), grain size (Kang et al., 2020), flag leaf trait (Wang et al., 2022). In this study, we combined these two approaches to characterize seedling salt tolerance in 295 materials and 189 RIL lines and obtained fifteen and six major QTLs, respectively. Comparing these genes to the findings of earlier research, they were near or overlapped with the loci of several known QTLs/genes. Ammar et al. (2009) found a salt tolerance-associated QTL (*qNaLV-3.1*), and the lead SNP (Chr3_25974535), detected by GWAS, were located within *qNaLV-3.1*. A QTL (*qRFW-6*) associated with the radicle fresh weight under salt stress was discovered by Mardani et al. (2014) on chromosome 6, and it contained the lead SNPs Chr6_19195187, Chr6_12954487, and Chr6_15555901 that were identified by GWAS. Atefeh et al. (2013) found a QTL (*qRL-10b*) on chromosome 10 associated with root length under osmotic stress, where the lead SNP (Chr10_4241399) identified by GWAS was located. The lead SNP (Chr12_19562318) detected by GWAS was located within the QTL (*qSNC-12*) for the concentrations of Na^+^ in shoots under salt stress detected by Zheng et al. (2015) using linkage analysis, and was close to the gene regulating iron distribution, *OsbHLH133* (Wang et al., 2013). The QTL *qRRN6* contained known genes *OsCYP19-4* (Yoon et al., 2016), which has peptidyl-prolyl cis-trans isomerase activity, and, *OsCYP19-4* overexpression plants enhance cold tolerance. Meanwhile, the GWAS identified the lead SNP (Chr2_22340368), which is located within *qRRL2* and *qRRDW2* identified by linkage analysis, both within the QTL (*qNAK-2*) associated with salt tolerance identified by Yao et al. (2005).

We found that *LOC_Os02g36880*, a NAC transcription factor, contains an apical meristem structural domain. NAC transcription factors have a role in regulating abiotic stress tolerance, leaf senescence, lateral root growth, programmed cell death, etc (Huysmans et al., 2018; Xia et al., 2018; Zhang et al., 2018; Singh et al., 2021; Wang et al., 2021). For example, the *OsNAC106* gene is a NAC transcription factor that negatively regulates salt tolerance, and the *osnac106* deletion mutant is more salinity tolerant than normal plants (Sakuraba et al., 2015). Nakashima et al. (2007) found that transgenic overexpressing *OsNAC6* had increased tolerance to high salt and drought stresses. Hong et al. (2016) identified *ONAC022* as a transcriptional activator that positively regulates salt tolerance by modulating abscisic acid signaling, and overexpression of *OsNAC022* transgenic plants were more salt-tolerant, accumulating less Na^+^ in roots and shoots than wild-type. It was shown that the NAC family of transcription factors is crucial in the study of salt tolerance.

In the previous study, Fang et al (Fang et al., 2014). found that the expression level of *LOC_Os02g36880* was significantly reduced under drought and increased under high salt stress. Wang et al. (2023) combined metabolomic and transcriptomic profiles and found that *LOC_Os02g36880* may be associated with low-nitrogen tolerance. The gene *LOC_Os02g36880* was determined to be the most likely functional gene linked to salt tolerance. Seedlings of the CRISPR/Cas9 mutant of the *LOC_Os02g36880* gene were substantially more saline-tolerant than wild-type, demonstrating that *LOC_Os02g36880* negatively regulates salt tolerance. We combined with haplotype analysis and found that CD belongs to Hap1 (AC) and WD20342 belongs to Hap2 (GA), as WD20342 is more salt-tolerant than CD, indicating that Hap2 belongs to the dominant salt tolerant haplotype. This provides a theoretical basis for breeding new salt-tolerant rice varieties and is essential for improving the utilization efficiency of saline land.

## Data Availability

The original contributions presented in the study are included in the article/**Supplementary Material**. Further inquiries can be directed to the corresponding author.

## Supplementary material

Supplementary Figure 1 Phenotypes of CD and WD20342 seedlings under control and salt stress. **(A)** Under control conditions. **(B)** Under salt stress. Bar = 5 cm.

Supplementary Figure 2 Linkage analysis results of RIL lines derived from CD/WD20342.

Supplementary Figure 3 Expression patterns of the other eight genes under normal growth and salt stress.

Supplementary Figure 4 The gene structure and sequence difference analysis of *LOC_Os02g36880*. **(A)** The gene structure of *LOC_Os02g36880*. **(B)** The sequence difference analysis of *LOC_Os02g36880* in WD20342, CD, and Nipponbare.
