# Supplementary figures and images for: GWAS combined with linkage analysis reveals major QTLs and candidate genes of salt tolerance in Japonica rice seedlings
Source: Front Plant Sci. 2024 Nov 1;15:1462856. doi: 10.3389/fpls.2024.1462856 (PMC11563981; doi:10.3389/fpls.2024.1462856)

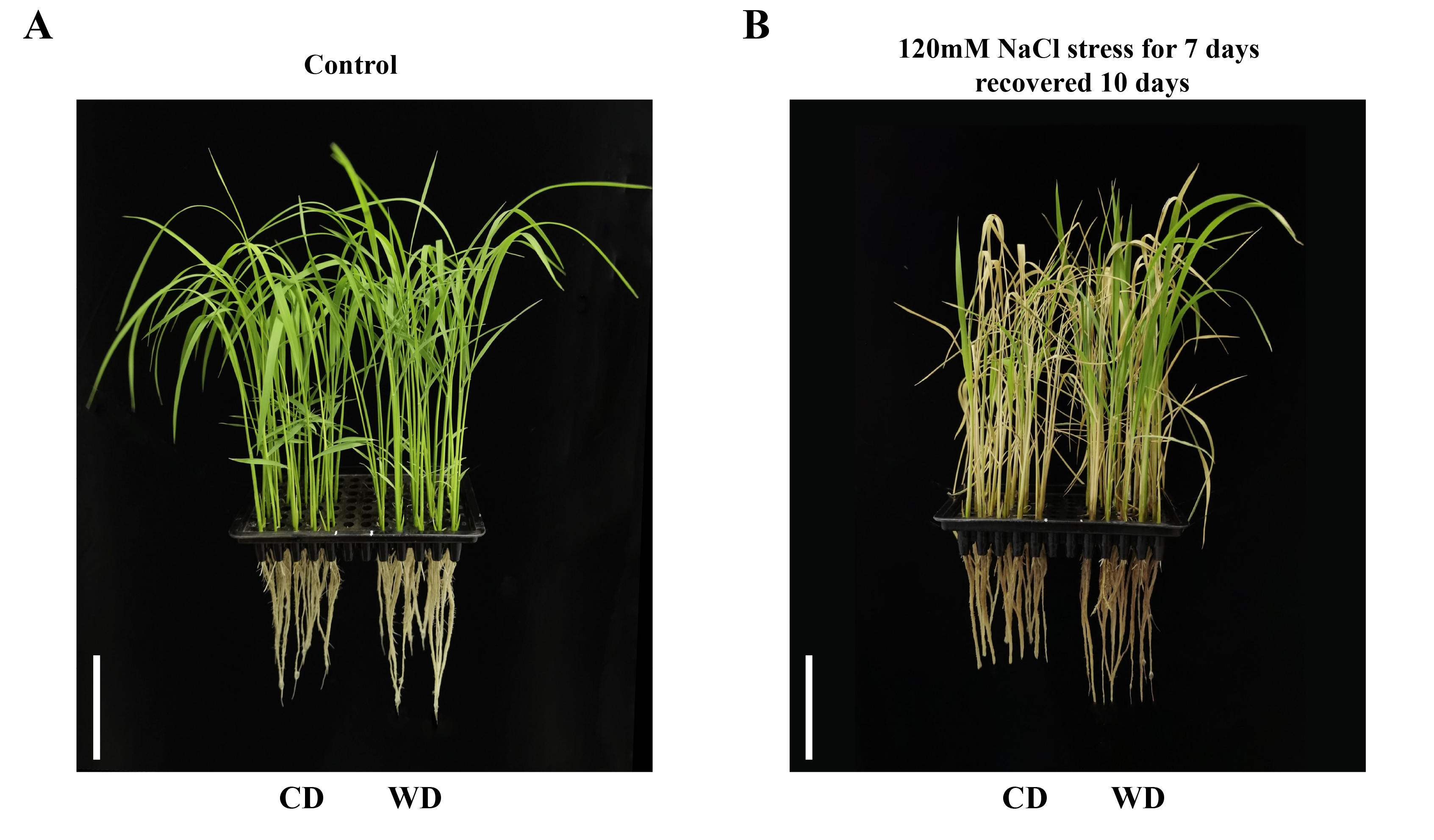

Supplement: Supplementary Figure 1 — Phenotypes of CD and WD20342 seedlings under control and salt stress. (A) Under control conditions. (B) Under salt stress. Bar = 5 cm. [file Image1.tif]

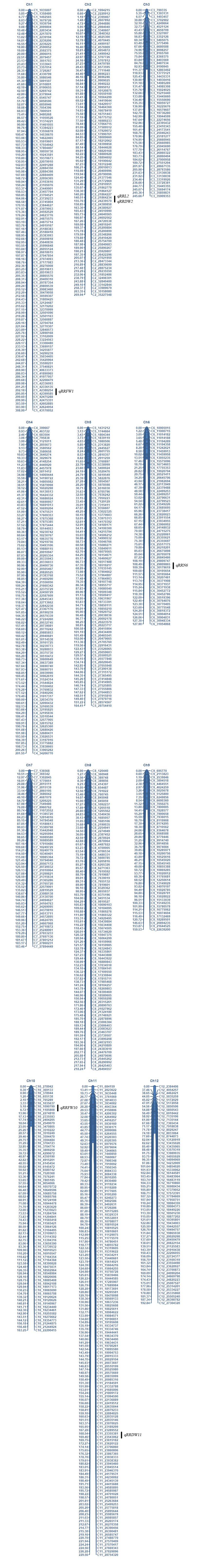

Supplement: Supplementary Figure 2 — Linkage analysis results of RIL lines derived from CD/WD20342. [file Image2.tif]

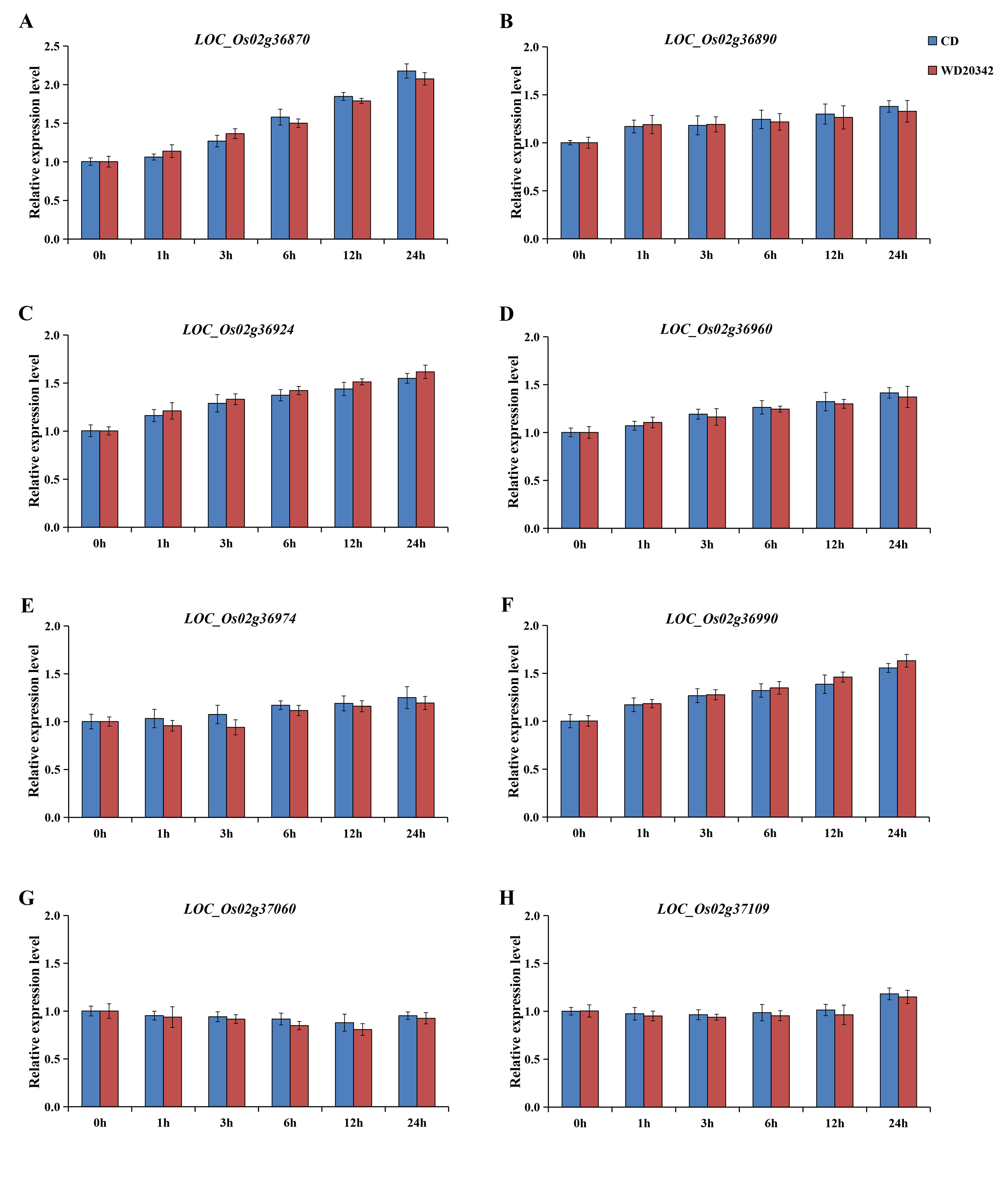

Supplement: Supplementary Figure 3 — Expression patterns of the other eight genes under normal growth and salt stress. [file Image3.tif]

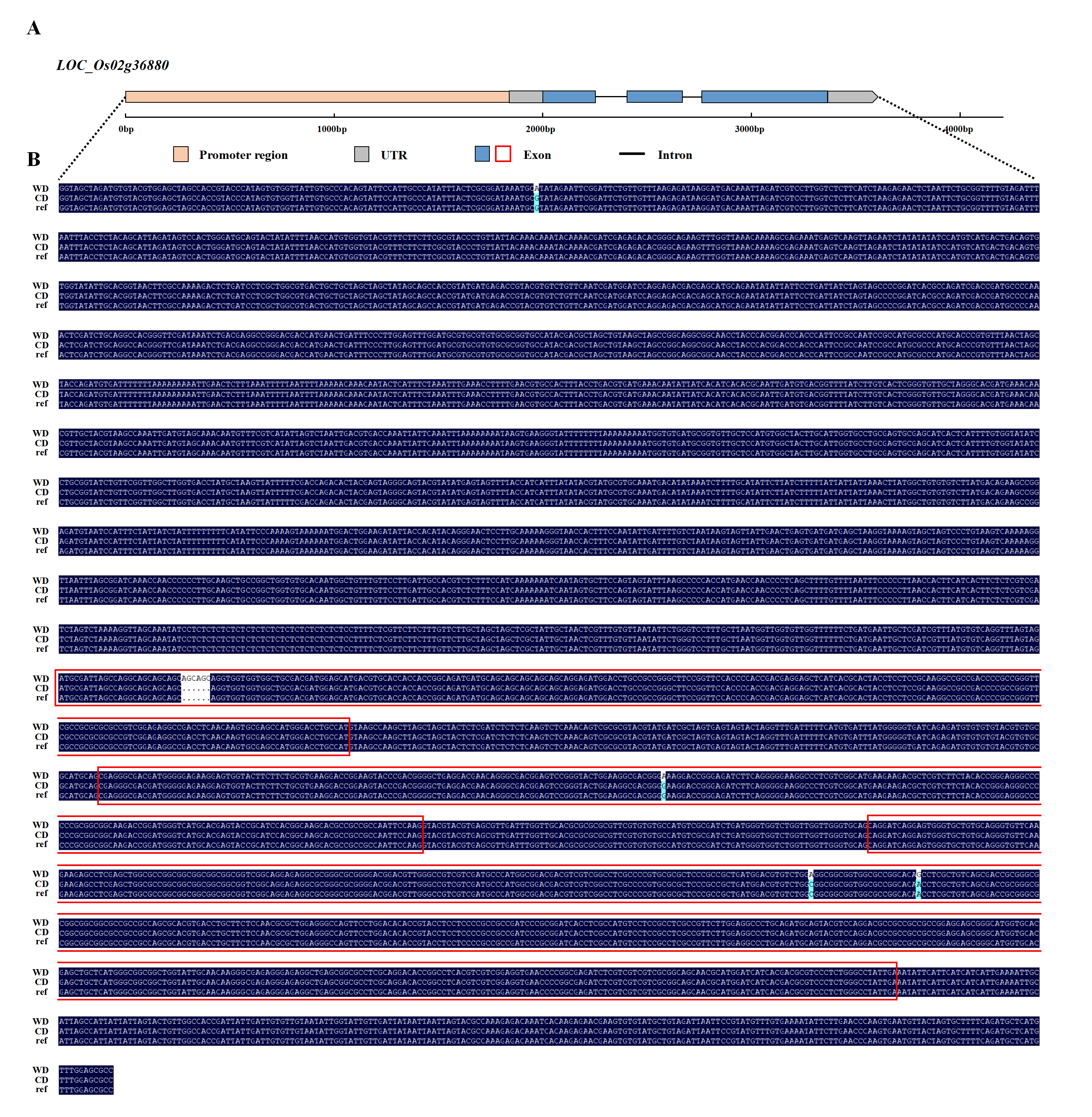

Supplement: Supplementary Figure 4 — The gene structure and sequence difference analysis of LOC_Os02g36880. (A) The gene structure of LOC_Os02g36880. (B) The sequence difference analysis of LOC_Os02g36880 in WD20342, CD, and Nipponbare. [file Image4.tif]
